# Prognostic stratification based on the levels of tumor-infiltrating myeloid-derived suppressor cells and PD-1/PD-L1 axis in locally advanced rectal cancer

**DOI:** 10.3389/fonc.2022.1018700

**Published:** 2022-10-25

**Authors:** Yu Jin Lim, Jaemoon Koh, Minji Choi, Sehui Kim, Eui Kyu Chie

**Affiliations:** ^1^ Department of Radiation Oncology, Kyung Hee University College of Medicine, Kyung Hee University Medical Center, Seoul, South Korea; ^2^ Department of Pathology, Seoul National University College of Medicine, Seoul, South Korea; ^3^ Medical Science Research Institute, Kyung Hee University Medical Center, Seoul, South Korea; ^4^ Department of Radiation Oncology, Seoul National University College of Medicine, Seoul, South Korea; ^5^ Institute of Radiation Medicine, Medical Research Center, Seoul National University, Seoul, South Korea

**Keywords:** rectal neoplasms, myeloid-derived suppressor cells, PD-1, PD-L1, CD8, risk factors, prognosis

## Abstract

**Background:**

Although rectal cancer remains somewhat sanctuary to the contemporary immunotherapy, there is increasing knowledge on clinical implications of anti-tumor immunity. This study evaluated the prognostic relevance of two immune-inhibitory functions, myeloid-derived suppressor cells (MDSCs) and programmed cell death-1 (PD-1)/programmed death-ligand 1 (PD-L1) axis.

**Methods:**

Study cohort is comprised of 165 patients with locally advanced rectal cancer who underwent neoadjuvant chemoradiotherapy followed by definitive resection. Using postsurgical tissue microarrays, the number of MDSCs, PD-1^+^/CD8^+^ tumor-infiltrating lymphocyte (TIL) ratio, and PD-L1 expression scores in stromal immune cells and tumor cells were assessed.

**Results:**

Positive correlation was observed between the PD-1^+^/CD8^+^ TIL ratio and number of MDSCs (*P* < 0.001). The greater the immune infiltrates, the higher the PD-L1 immune cell score (*P* < 0.001). MDSC^High^, PD-1^+^/CD8^+^ TIL^High^, PD-L1 immune cell score^Low^, and PD-L1 tumor H-score^High^ were associated with worse disease-free survival (DFS) (*P* < 0.001, *P* = 0.042, 0.047, and *P* < 0.001, respectively). To integrate the adverse effects of MDSC^High^, PD-1^+^/CD8^+^ TIL^High^, and either PD-L1 immune cell score^Low^ (set I) or tumor H-score^High^ (set II), prognostic risks were stratified according to the number of factors: 0, 1, and 2−3 (*P* < 0.001 for I and II). On multivariate analyses, patients with multiple risk factors for set I and II had worse prognosis (*P* < 0.001; 2−3 vs. 0 for models I and II), and the two prognostic models had acceptable predictability.

**Conclusion:**

In this study, integration of the prognostic impact of MDSCs and PD-1/PD-L1 stratified the long-term risks of patients with locally advanced rectal cancer. Thus, further exploration could be focused to the identified subset of patients carrying worse prognosis, where potential benefits could be derived by targeting the two components contributing to the immunosuppressive microenvironment.

## Introduction

Colorectal cancer ranks as the 3^rd^ most common malignancy and is also the 3^rd^ cause of cancer mortality (1). Despite increasing knowledge on treatment modalities in locally advanced rectal cancer, such as total mesorectal excision, neoadjuvant chemoradiotherapy (CRT), and more recently, total neoadjuvant therapy, further diagnostic and therapeutic efforts are required to improve oncologic outcomes.

In the contemporary era, cancer immunotherapy, which blocks the programmed cell death-1 (PD-1)/programmed death-ligand 1 (PD-L1) axis, is a novel treatment approach in a variety of malignancies. However, the checkpoint inhibitors have not gained sufficient grounds in colorectal cancer. Especially in rectal cancer, a limited proportion of microsatellite instability (MSI) tumors may be one of top concerns (2). Nevertheless, there is still a therapeutic potential of this approach, because the cytotoxic neoadjuvant CRT, the standard treatment of locally advanced rectal cancer, can be a source of neoantigens, converting tumors from “cold” to “hot” status (3). To identify patients who are likely to benefit from the PD-1/PD-L1-inhibiting strategy, it is mandatory to comprehensively analyze the individual expression levels of related immune cell markers in the tumor microenvironment.

Myeloid-derived suppressor cells (MDSCs) are a heterogeneous group of immature myeloid cells that play important roles in tumor immune evasion, progression, and metastasis (4). MDSCs in the tumor microenvironment suppress the proliferation and activation of cytotoxic T cells, contributing to dysfunctional anti-tumor immunity, and consequently, the immunosuppressive tumor contexture (5). Although the clinical implications of relevant immune-inhibitory mechanisms have been suggested (6), tumor-infiltrating MDSCs have not been commonly analyzed in daily practice because of their heterogeneous characteristics and the equivocal definition of the cell subset. For the practical use of MDSC as an immunotherapeutic target, more clinical evidence is needed to ascertain their predictive and prognostic potential.

Here, we focused on the major components contributing to the immunosuppressive tumor microenvironment, MDSCs and PD-1/PD-L1 axis, as potential immunotherapeutic targets in rectal cancer. Using double immunofluorescence (IF), tumor-infiltrating MDSCs were identified within the rectal cancer tissues treated with CRT. The expression levels of the PD-1/PD-L1 axis were comprehensively assessed in tumor and stromal areas. Resulting prognostic risk stratification may provide initial insights into the dual-targeting of these two immune-inhibitory components for patients with locally advanced rectal cancer.

## Materials and methods

### Study population

Patients with locally advanced rectal cancer who underwent neoadjuvant CRT followed by total mesorectal excision were retrospectively analyzed. The inclusion criteria were as follows: (1) pathologically confirmed diagnosis of adenocarcinoma; (2) clinical stage ≥ cT3 and/or cN1−2; (3) no distant metastasis at diagnosis; (4) CRT regimen with conventional fractionation; (5) availability of postsurgical tumor specimens; and (6) completion of CRT followed by surgery. This study was approved by the institutional review board (no. 1708-052-876).

Tumor regression grade (TRG) was assessed using the Dworak system: 0 (no regression) to 4 (complete regression) (7). Microsatellite status was assessed by fluorescent multiplex polymerase chain reaction with five markers (BAT-25, BAT-26, D5S346, D17S250, and D2S123) recommended by the National Cancer Institute workshop. Cases with two or more markers were assessed as microsatellite instability-high (MSI-H), and cases with one of the markers were assessed as MSI-low (MSI-L). When all markers were stable, the cases were diagnosed as microsatellite-stable (MSS). The treatment protocol used for CRT has been reported previously (8).

### Immunofluorescence staining

All postsurgical specimens were retrieved from the pathology archive of Seoul National University Hospital. Tissue microarray (TMA) blocks were made with 4-mm cores of formalin-fixed paraffin-embedded tumor tissues consisting of two representative cores of the invasive front for each case. The TMA slides were soaked in xylene twice for 10 min each. The slides were rehydrated with 5-min washes in 100% ethanol twice, followed by 5-min washes in 95-90-80-70% ethanol each and 5-min rinse under streaming tap water. Antigen retrieval was then performed using sodium citrate buffer (pH 6.0) for 10 min at 120°C. For permeabilization, the slides were washed with phosphate buffered saline (PBS) three times and 15-min incubation in 0.2% Triton × 100 in PBS. Subsequently, nonspecific antigens were blocked using 0.2% bovine serum albumin in PBS for 1 h. For the double-staining of CD11b and CD33, a cocktail of primary antibodies including anti-CD11b (#NB110-89474, Novus Biologics; 1:800) and anti-CD33 (#MAB11371-100, R&D systems; 1:500), was incubated in blocking solution overnight at 4°C. The following day, the slides were incubated with Alexa 488-conjugated secondary antibody (#A-11034, Invitrogen; 1:200) and Alexa 546-conjugated secondary antibody (#A-11003, Invitrogen; 1:200) for 1 h at room temperature. Finally, the nuclei were stained with 4,6-diamidino-2-phenylindole (#62247; Thermo Fisher Scientific; 1:1000) for 10 min at room temperature and mounted with a mounting reagent (#E01-100, GBI Labs).

### Immunohistochemical staining

Immunohistochemical (IHC) staining of PD-1, CD8, and PD-L1 was performed as previously described (9). The staining antibodies used were rabbit anti-PD-1 (#ab137132, Abcam; 1:300), rabbit anti-CD8 (#MA5-14548, Thermo Fisher Scientific; 1:100), and rabbit anti-PD-L1 (#13684, Cell Signaling Technology; 1:30). Human tonsil and placental tissues were used as positive control.

### Pathological evaluation

The number of MDSCs was assessed using double IF for CD11b and CD33. The unstained slides were serially produced from the tissue microarrays, and one sheet was stained with H&E and another sheet was used for immunofluorescence staining. Then, the corresponding locations and features focusing the same cells were considered and analyzed to evaluate the presence and the number of MDSCs. The MDSC was defined as CD11b^+^ and CD33^+^ with myeloid cell features: a selective reading of cells having the shape of a typical myeloid cell that is larger than the size of a lymphocyte and has a cytoplasm to some extent. The specimens were examined under a fluorescence microscope (Zeiss LSM700; Zeiss, Jena, Germany) equipped with a high-resolution camera (AxioCam; Zeiss, Jena, Germany). All high-resolution images of the TMA slides were acquired and examined using a Zeiss LSM700 confocal microscope (Zeiss, Jena, Germany) at 20× and 40× magnifications in the fluorescent mode. Images were captured using a ZEN 2012 (Zeiss, Jena, Germany). For each sample, at least three high-power fields showing representative tumor areas were selected and photographed. With each of captured high power field images, the numbers of MDSCs were counted manually by experienced pathologists (J.K. and S.K.).

PD-L1 expression in immune cells and tumor cells was analyzed separately by modifying a previously used approach (10, 11). For tumor cells, membranous expression was assessed. For immune cells, the PD-L1 expression on tumor-infiltrating immune cells were assessed based on the membranous and/or cytoplasmic expression. To evaluate the expression level, PD-L1 scores for immune cells and tumor cells, hereafter referred to as immune cell score and H-score, respectively, were determined by multiplying the percentage of PD-L1-positive cells (0−100%) with staining intensity (0−3), thus ranging from 0 to 300. The IHC slides of PD-1 and CD8 were scanned using AperioScanScope (Aperio Technologies, CA, USA). The total percentage of positive cells was scored from 2+ to 3+ for each case using the Aperio nuclear IHC algorithm. Based on the formula defined as the total number of 2+ to 3+ cells divided by area (mm^2^), the density values of PD-1^+^ and CD8^+^ tumor-infiltrating lymphocytes (TILs) were automatically enumerated. As verified in previous studies (9, 12), the PD-1^+^/CD8^+^ TIL ratio was calculated for each patient to estimate the relativeness of immune-exhaustion to cytotoxic effector activity. For overall pathological evaluation, two pathologists (J.K. and S.K.) independently reviewed the IHC and IF slides, blinded to the clinical information.

### Statistical analysis

To compare continuous variables between two groups, Student’s t-test or Mann-Whitney U test was used. To compare multiple groups, one-way analysis of variance followed by *post-hoc* Tukey’s test or Kruskall Wallis test was used for parametric or non-parametric variables, respectively. The maximal chi-square method based on the events of tumor recurrence or death was used to determine optimal cutoff values: < 3 vs. ≥ 3, ≤ 0.1725 vs. > 0.1725, ≤ 20 vs. > 20, and ≤ 200 vs. > 200 for low vs. high levels of MDSCs, PD-1^+^/CD8^+^ TIL ratio, stromal PD-L1 immune cell score, and tumor PD-L1 H-score, respectively. Pearson’s chi-square or Fisher’s exact test was used to evaluate the relationships between two variables as appropriate. For survival analysis, disease-free survival (DFS) was defined as the period between the start date of CRT and the overall events of death or tumor recurrence. Overall survival (OS) was calculated by considering the time period for death events. Kaplan-Meier analysis with log-rank test was used to compare survival outcomes according to clinicopathological variables. Multivariate analyses for independent prognostic factors were conducted using Cox proportional hazards models. The Harrell C statistic method was used to estimate the discriminatory ability of a prognostic model based on time-to-event survival data. The likelihood ratio test was used to compare the c-index values. Statistical significance was set at *P* < 0.05. All statistical analyses were performed using SPSS 18 (IBM, NY, USA) and R software (version 3.6.1; R Development Core Team; http://www.r-project.org).

## Results

### Characteristics of study population

The patient, tumor, and treatment-related characteristics of the study population (n = 165) are summarized in **Supplementary Table 1**. With a median age of 62 years (range, 29−86 years), 70% of the patients were male (n = 116). Distal end of the primary tumor location ranged from 0 cm to 12 cm from the anal verge. For clinical stage, patients with cT3 and/or cN1−2 were most frequent (84% for both). Pathologic finding after the CRT revealed 45% (n = 74) with ypT3 and 28% (n = 47) with ypT2 tumors. No nodal involvement was reported in 77% of the specimen (n = 127). For pathologic TRG, 24% (n = 40), 36% (n = 59), and 38% (n = 62) had Dworak TRG of 3−4 (near complete or complete), 2 (moderate), and 0−1 (minimal to no), respectively. Lymphatic, vascular, and perineural invasion were observed in 12% (n = 20), 4% (n = 6), and 9% (n = 15) of patients, respectively. The median carcinoembryonic antigen levels (ng/mL) prior to CRT, after CRT and prior to surgery, and after surgery were 2.3 (range, 0.0−300.0), 1.6 (range, 0.0−42.9), and 1.1 (0.0−7.2), respectively. MSS tumor was much more prevalent with 63%, whereas MSI-L or MSI-H was observed in 6%. The median time interval between completion of CRT and surgery was 53 days (range, 38−111 days). Majority of the patients (94%) underwent sphincter preserving surgery. Nearly 90% of the patients underwent postoperative chemotherapy.

### MDSCs and PD-1/PD-L1 axis

The median H-score and immune cell scores of PD-L1 were 140 (range, 0−300) and 120 (range, 0−300), respectively. Based on the median cell density of PD-1^+^ and CD8^+^ TILs of 28.2 cells/mm^2^ (range, 0.0−191.2) and 727.4 cells/mm^2^ (range, 0.0−2913.8), respectively, the median value of the PD-1^+^/CD8^+^ TIL ratio was 0.040 (range, 0.002−0.777). The median enumerated count of CD11b^+^CD33^+^ MDSCs was three (range, 0−16).

**Figure 1:**
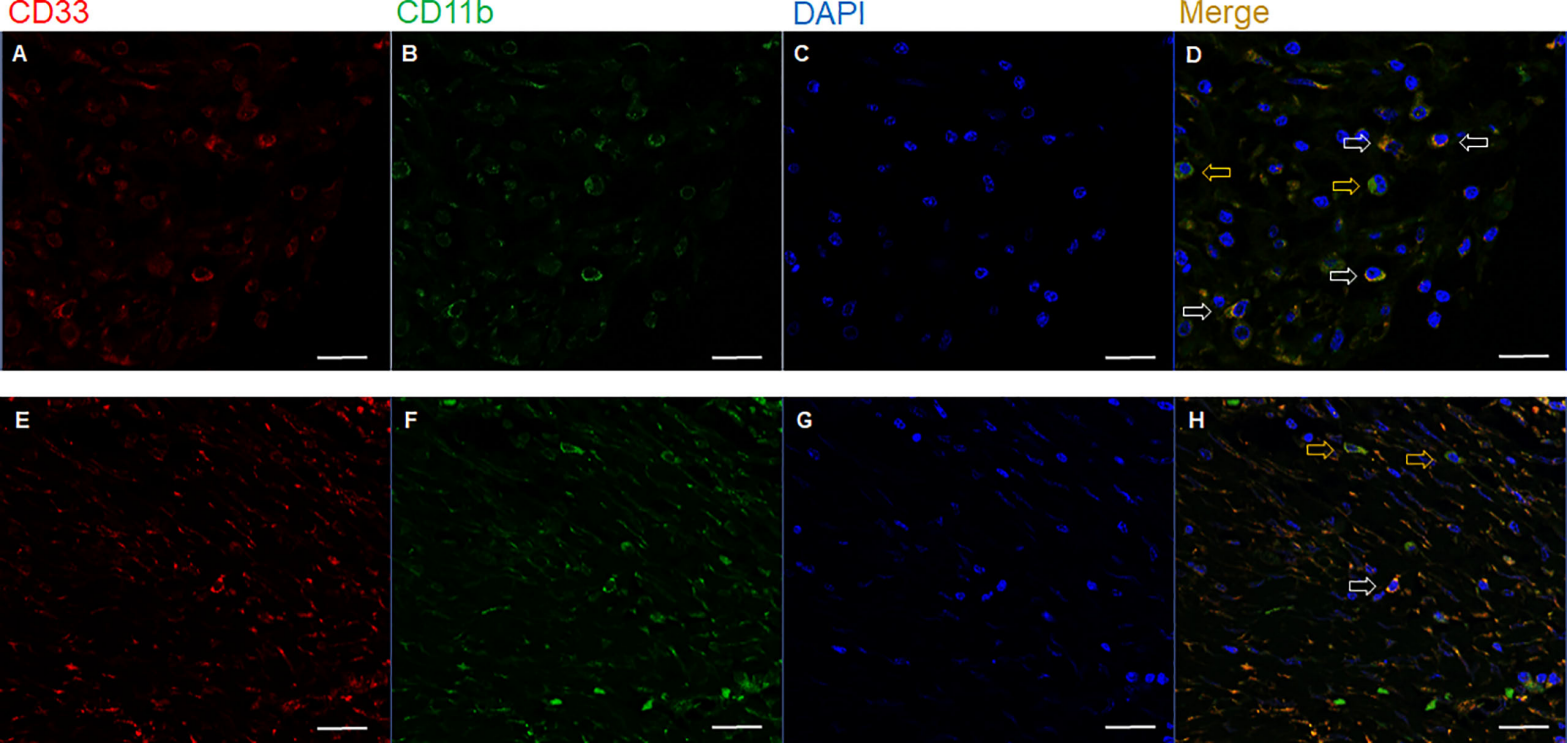
Representative immunofluorescence images of two cases with **(A−D)** high (upper row) and **(E−H)** low (lower row) number of tumor-infiltrating MDSCs. Based on the expression of **(A, E)** CD33, **(B, F)** CD11b, and **(C, G)** DAPI, **(D, H)** the merged triple immunofluorescence results were assessed. Cells with yellow color were merged from red and green. yellow arrows: CD11b^+^ CD33^-^ cells; white arrows: CD11b^+^ CD33^+^ cells; Scale bar: 20 μm. MDSC, myeloid-derived suppressor cell; DAPI, 4,6-diamidino-2-phenylindole.

**Figure 2:**
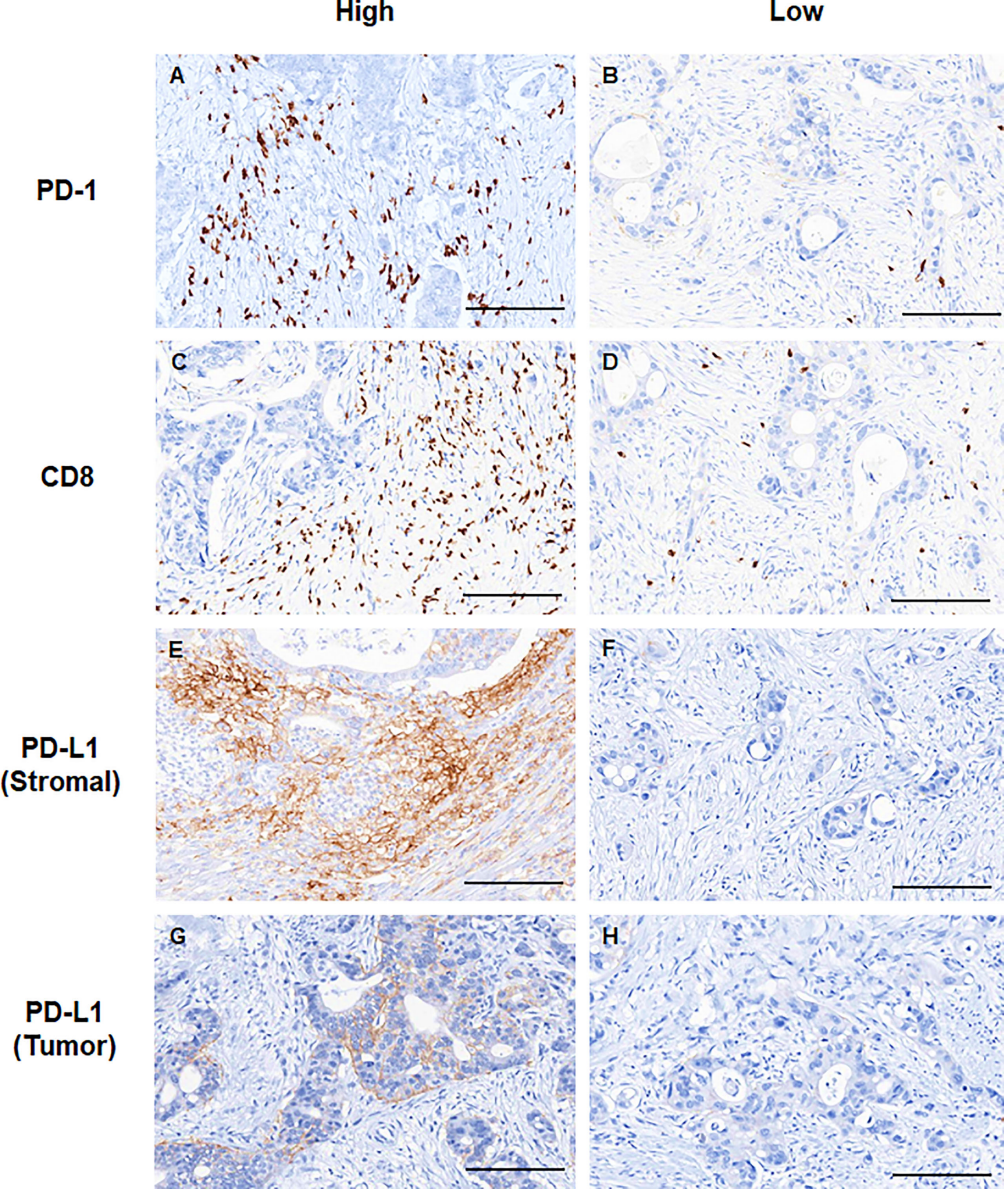
Representative immunohistochemical images showing high (*left column*) and low (*right column*) expression levels of **(A, B)** PD-1, **(C, D)** CD8, **(E, F)** stromal PD-L1, and **(G, H)** tumor PD-L1. Scale bar: 100 μm. PD-1, programmed cell death-1; PD-L1, programmed death-ligand 1.

**Figure 3:**
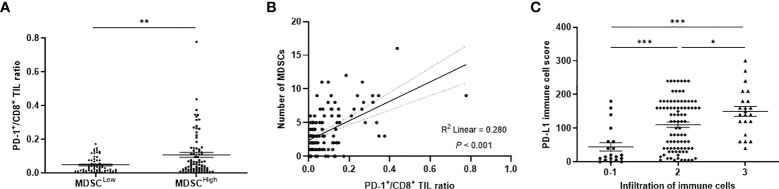
**(A)** PD-1^+^/CD8^+^ TIL ratio values according to low and high levels of MDSCs. **(B)** Simple linear regression analysis representing a positive correlation between the PD-1^+^/CD8^+^ TIL ratio and the number of MDSCs. **(C)** Distribution of PD-L1 immune cell score according to the infiltration level of overall immune cells. PD-1, programmed cell death-1; TIL, tumor-infiltrating lymphocyte; MDSC, myeloid-derived suppressor cell; PD-L1, programmed death-ligand 1. *P < 0.05, **P < 0.01, and ***P < 0.001.

Representative slide views of high and low levels of MDSCs and PD-1/PD-L1 axis are shown in **Figure 1** (MDSCs) and **Figure 2** (PD-1, CD8, and PD-L1 on stromal immune cells and tumor cells). The number of patients in each group is listed in **Supplementary Table 2**. The mean PD-1^+^/CD8^+^ TIL ratio was higher in the MDSC^High^ group than in the MDSC^Low^ group (*P* = 0.007) (**Figure 3A**), and a positive correlation was observed between the number of MDSCs and the PD-1^+^/CD8^+^ TIL ratio (linear R^2^ = 0.280; *P* < 0.001) (**Figure 3B**). Intratumoral immune cell infiltration was graded from 0 to 3 in increasing order, and there was a positive correlation between the overall immune infiltrates and the mean stromal PD-L1 immune cell score (*P* < 0.001 for overall, comparing grades 0−1, 2, and 3) (**Figure 3C**). The number of MDSCs was higher in older patients (*P* = 0.047) and in tumors with minimal TRG (*P* = 0.027). The higher level of PD-L1 H-score was significantly associated with a higher proportion of ≥ ypT3 (*P* = 0.011), ypN+ (*P* = 0.003), no downstaging of T (*P* = 0.046) or N (*P* = 0.014), and minimal tumor regression (*P* = 0.053). The lower PD-L1 H-score was related with no lymphatic (*P* = 0.003) and perineural invasion (*P* = 0.059) (**Supplementary Table 3**).

### Survival analysis and prognostic models

**Figure 4:**
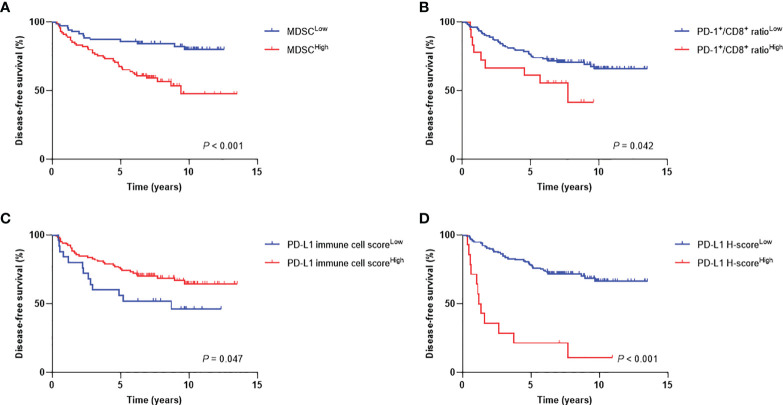
Disease-free survival curves according to high and low levels of **(A)** MDSCs, **(B)** PD-1^+^/CD8^+^ TIL ratio, **(C)** PD-L1 immune cell score, and **(D)** PD-L1 H-score. MDSC, myeloid-derived suppressor cell; PD-1, programmed cell death-1; TIL, tumor-infiltrating lymphocyte; PD-L1, programmed death-ligand 1.

The median follow-up duration was 5.9 years (range, 0.5−13.2 years). The 7-year DFS and OS rates were 71% and 78%, respectively. When patients were stratified into low or high levels, patients with MDSC^High^ (*P* < 0.001), PD-1^+^/CD8^+^ ratio^High^ (*P* = 0.042), PD-L1 immune cell score^Low^ (*P* = 0.047), and PD-L1 H-score^High^ (*P* < 0.001) showed inferior DFS (**Figure 4**). There were also significant differences in OS according to MDSC level (*P* = 0.001), PD-1^+^/CD8^+^ TIL ratio (*P* = 0.046), and PD-L1 H-score (*P* = 0.001) (**Supplementary Figure 1**). The 7-year DFS rates of the MDSC^Low^ vs. MDSC^High^ and PD-1^+^/CD8^+^ ratio^Low^ vs. PD-1^+^/CD8^+^ ratio^High^ groups were 84% vs. 59%, and 71% vs. 56%, respectively. Regarding PD-L1 expression levels, the 7-year DFS rates of low vs. high stromal PD-L1 immune cell score and low vs. high tumor PD-L1 H-score were 52% vs. 70% and 72% vs. 21%, respectively.

**Figure 5:**
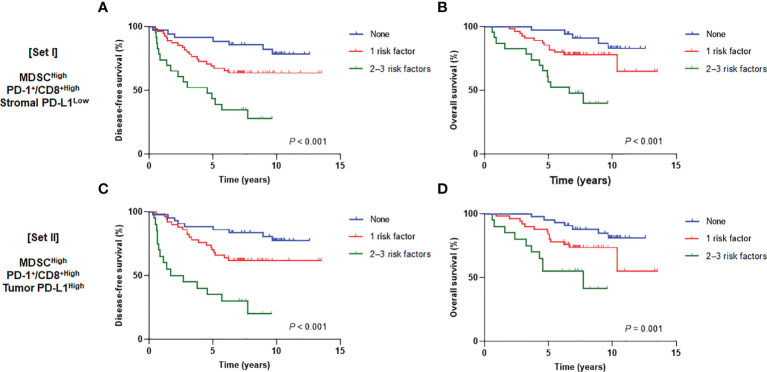
Disease-free survival (*left*) and overall survival (*right*) curves according to the number of risk factors from **(A, B)** set I (MDSCs, PD-1^+^/CD8^+^ TIL ratio, and stromal PD-L1) and **(C, D)** set II (MDSCs, PD-1^+^/CD8^+^ TIL ratio, and tumor PD-L1). MDSC, myeloid-derived suppressor cell; PD-1, programmed cell death-1; TIL, tumor-infiltrating lymphocyte; PD-L1, programmed death-ligand 1.

We then assumed that the MDSC^High^, PD-1^+^/CD8^+^ ratio^High^, stromal PD-L1 immune cell score^Low^, and tumor PD-L1 H-score^High^ could be discrete risk factors relevant to the status of the tumor-immune microenvironment. Regarding significant association between stromal and tumor PD-L1 scores, two types of combination sets integrating the prognostic effects of MDSCs and the PD-1/PD-L1 axis were defined as follows: 1) MDSCs, PD-1^+^/CD8^+^ TIL ratio, and stromal PD-L1 immune cell score (combined set I) and 2) MDSCs, PD-1^+^/CD8^+^ TIL ratio, and tumor PD-L1 H-score (combined set II). When the study population was classified into three subgroups with 0, 1, and 2−3 risk factors, there were significant survival differences according to the risk levels in both sets I and II (*P* < 0.001 for DFS and OS in set I; *P* < 0.001 and *P* = 0.001 for DFS and OS, respectively, in set II) (**Figure 5**). In multivariate analyses of DFS, adjusting for other related covariates from univariate analyses (**Supplementary Table 4**), patients with single risk factor (hazard ratio [HR] 2.83, *P* = 0.058) and multiple risk factors (HR 9.12, *P* < 0.001) for the set I had worse outcomes (model I). Similar results were also observed in model II (HR 3.19, *P* = 0.027 and HR 10.66, *P* < 0.001 for single and multiple risk factors for the set II, respectively) (**Table 1**). The existence of single (*P* = 0.021 and 0.017 for models I and II, respectively) and multiple risk factors (*P* < 0.001 and *P* = 0.005 for models I and II, respectively) were also associated with inferior OS (**Table 2**).

**Table 1:** Cox proportional-hazards regression models for disease-free survival.

Variables	DFS model (I)		DFS model (II)	
	HR (95% CI)	*P*	HR (95% CI)	*P*
ypT stage				
T0–2	Ref		Ref	
≥ T3	1.69 (0.75−3.78)	0.203	1.96 (0.85−4.50)	0.116
ypN stage				
N0	Ref		Ref	
N1–2	2.59 (1.11−6.09)	0.029	2.38 (0.96−5.89)	0.061
Dworak TRG				
3–4	Ref		Ref	
2	2.63 (0.31−22.49)	0.376	2.26 (0.25−20.29)	0.466
0–1	6.57 (0.80−53.89)	0.080	4.81 (0.55−41.80)	0.155
Lymphatic invasion				
No	Ref		Ref	
Yes	1.30 (0.53−3.21)	0.569	1.47 (0.61−3.53)	0.387
Perineural invasion				
No	Ref		Ref	
Yes	2.66 (0.86−8.20)	0.089	2.82 (0.85−9.41)	0.092
Pre-RT CEA				
Low	Ref		Ref	
High	2.07 (0.77−5.59)	0.150	2.86 (1.06−7.70)	0.038
Post-RT CEA				
Low	Ref		Ref	
High	1.18 (0.50−2.79)	0.707	0.75 (0.31−1.83)	0.523
Post-Op CEA				
Low	Ref		Ref	
High	0.65 (0.29−1.45)	0.292	0.63 (0.28−1.44)	0.276
Types of surgery				
Sphincter-preserving	Ref		Ref	
APR	1.58 (0.46−5.44)	0.467	1.61 (0.50−5.22)	0.425
MDSC^High^, PD-1^+^/CD8^+High^, and PD-L1 immune cell score^Low^				
None	Ref			
1 risk factor	2.83 (0.96−8.33)	0.058		
2−3 risk factors	9.12 (2.86−29.13)	< 0.001		
MDSC^High^, PD-1^+^/CD8^+High^, and PD-L1 H-score^High^				
None			Ref	
1 risk factor			3.19 (1.15−8.91)	0.027
2−3 risk factors			10.66 (3.38−33.57)	< 0.001

DFS, disease-free survival; HR, hazard ratio; CI, confidence interval; Ref, reference; TRG, tumor regression grade; RT, radiotherapy; CEA, carcinoembryonic antigen; APR, abdominoperineal resection; MDSC, myeloid-derived suppressor cell; PD-1, programmed cell death-1; PD-L1, programmed death-ligand 1.

**Table 2:** Cox proportional-hazards regression models for overall survival.

Variables	OS model (I)		OS model (II)	
	HR (95% CI)	*P*	HR (95% CI)	*P*
Age (years)				
≤ 62	Ref		Ref	
> 62	5.10 (1.90−13.67)	0.001	4.43 (1.71−11.49)	0.002
ypT stage				
T0–2	Ref		Ref	
≥ T3	2.22 (0.78−6.36)	0.137	2.11 (0.76−5.87)	0.154
ypN stage				
N0	Ref		Ref	
N1–2	5.96 (2.09−17.03)	0.001	4.44 (1.55−12.70)	0.005
Dworak TRG				
3–4	Ref		Ref	
2	2.38 (0.23−24.36)	0.466	2.00 (0.20−19.96)	0.555
0–1	6.92 (0.71−67.32)	0.096	5.90 (0.60−58.30)	0.129
Lymphatic invasion				
No	Ref		Ref	
Yes	0.48 (0.13−1.77)	0.270	0.68 (0.22−2.18)	0.522
Perineural invasion				
No	Ref		Ref	
Yes	6.87 (1.59−29.63)	0.010	5.18 (1.24−21.74)	0.025
Pre-RT CEA				
Low	Ref		Ref	
High	4.98 (1.39−17.93)	0.014	6.64 (1.95−22.54)	0.002
Post-RT CEA				
Low	Ref		Ref	
High	1.65 (0.60−4.54)	0.331	1.10 (0.41−2.94)	0.856
Post-Op CEA				
Low	Ref		Ref	
High	0.38 (0.14−1.03)	0.057	0.45 (0.16−1.22)	0.117
Types of surgery				
Sphincter-preserving	Ref		Ref	
APR	0.94 (0.21−4.21)	0.934	0.76 (0.18−3.19)	0.705
MDSC^High^, PD-1^+^/CD8^+High^, and PD-L1 immune cell score^Low^				
None	Ref			
1 risk factor	6.49 (1.32−31.88)	0.021		
2−3 risk factors	17.80 (3.76−84.24)	< 0.001		
MDSC^High^, PD-1^+^/CD8^+High^, and PD-L1 H-score^High^				
None			Ref	
1 risk factor			4.87 (1.33−17.78)	0.017
2−3 risk factors			7.07 (1.79−27.87)	0.005

OS, overall survival; HR, hazard ratio; CI, confidence interval; Ref, reference; TRG, tumor regression grade; RT, radiotherapy; CEA, carcinoembryonic antigen; Op, operation; APR, abdominoperineal resection; MDSC, myeloid-derived suppressor cell; PD-1, programmed cell death-1; PD-L1, programmed death-ligand 1.

### Comparison of prognostic models

The prognostic strength for each model was estimated with c-index values (**Table 3**). Relevant values of the model I were 0.788 and 0.877 for DFS and OS, whereas 0.796 and 0.849 for DFS and OS for the model II, respectively. Prognostic model II was more robust for DFS, whereas model I was better for OS (*P* < 0.001 for DFS and OS).

**Table 3:** Prognostic strength of the two models integrating the levels of MDSCs and PD-1/PD-L1 axis.

Prognostic model	C-index for DFS	C-index for OS
Model I including MDSCs, PD-1^+^/CD8^+^ ratio, and stromal PD-L1 score	0.788	0.877
Model II including MDSCs, PD-1^+^/CD8^+^ ratio, and tumor PD-L1 score	0.796	0.849

MDSC, myeloid-derived suppressor cell; PD-1, programmed cell death-1; PD-L1, programmed death-ligand 1; DFS, disease-free survival; OS, overall survival.

## Discussion

Based on the postsurgical specimens with long-term follow-up data, we demonstrated the prognostic relevance of the two major immunosuppressive components, MDSCs and the PD-1/PD-L1 immune checkpoint, in the tumor microenvironment. There was a significant positive correlation between the PD-1^+^/CD8^+^ TIL ratio and the number of MDSCs. The greater infiltration of overall immune cells was associated with higher stromal PD-L1 immune cell score. Furthermore, a higher number of MDSCs, higher PD-1^+^/CD8^+^ TIL ratio, lower stromal PD-L1 immune cell score, and elevated PD-L1 H-score of tumor cells were significantly associated with worse prognosis. Integrating the prognostic associations of MDSC^High^, PD-1^+^/CD8^+^ ratio^High^, and either PD-L1 immune cell score^Low^ or H-score^High^, patients were stratified according to the combined risk levels. To the best of our knowledge, this is the first study to evaluate the combined prognostic potential of tumor-infiltrating MDSCs and PD-1/PD-L1 axis in patients with locally advanced rectal cancer treated with neoadjuvant CRT.

Despite widespread heap of immunotherapy in oncology society in recent years, rectal cancer remains somewhat sanctuary to this trend with somewhat limited, if not negligible use of the contemporary PD-1/PD-L1 blockade. The limitation in rectal cancer is that MSS tumors account for a far greater portion of patients. Another component of immune-inhibitory mechanisms, MDSCs, are a heterogeneous group of immature myeloid cells that act as key mediators in assisting tumors to escape immune surveillance and cancer progression or metastasis. The clinical implications of MDSCs have been evaluated in various malignancies (13–16), but the heterogeneity within the cell population and relatively equivocal phenotyping were major obstacles for application in clinical practice (17). As immune-inhibitory mechanisms differ depending on cancer types, it is essential to analyze MDSCs for each malignancy of interest. Taken together, to ascertain the potential immunotherapeutic targets in locally advanced rectal cancer, we explored the prognostic associations of MDSC infiltration in combination with the expression levels of the PD-1/PD-L1 axis in the tumor microenvironment.

In this study, long-term outcome risks of individual patient were effectively stratified according to the number of prognostic factors. Based on these results, we inferred that the adverse prognostic effect from each variable might be added to each other when the discrete immune-inhibitory components work together in the tumor microenvironment. Activated MDSCs express PD-L1 that interacts with its counterpart, PD-1 on activated T cells, which leads to the exhaustion of effector T cells (18). Regulatory T cells also stimulate PD-L1 expression on MDSCs, thus suggesting the co-operating immunosuppressive network *via* PD-1/PD-L1 activity (19). Regarding the mechanistic and clinical significance of tumor-infiltrating MDSCs, several clinical trials have investigated potential benefits of dual-targeting of MDSCs and immune checkpoints (20–23). Accordingly, current analysis provides the initial insights into the combined targeting strategies, and nominate the subgroup of patients with locally advanced rectal cancer who might benefit from the combined immunotherapy.

PD-L1 expression on tumor-infiltrating immune cells per se indicates immune-inhibitory potential in the tumor milieu. However, this aspect is not directly linked to the status of the tumor-immune microenvironment owing to the dynamic characteristics of immunologic equilibrium (24). Our long-term outcome analysis exhibited worse prognosis with stronger tumor PD-L1 expression (H-score), whereas high PD-L1 expression in stromal immune cells (immune cell score) was associated with extended survival. In the tumor-immune microenvironment, the PD-L1 expression is particularly frequent in CD68^+^ macrophages in comparison to other immune cell types (25). In a variety of cancers, one potent inducer is IFN-γ. Given pro-inflammatory conditions, tumor-specific CTLs release IFN-γ, which induces the PD-L1 expression in cancer cells (26). Regarding its expression in tumor-infiltrating immune cells, mostly tumor-associated macrophages and MDSCs, several important mediators have been suggested. A recent investigation reported that the inflammatory cytokine IL-1β secreted by M1 macrophages induce the expression of PD-L1 *via* transcription factors IRF1 and P65 (27). Another study suggested that the COX2/mPGES1/PGE2 pathway regulates PD-L1 expression in tumor-associated macrophages and MDSCs (28). Especially under cytotoxic therapies, such as radiotherapy and chemotherapy, the increased tumor antigen load from the cell-killing effects contributes to more inflamed tumor status.

In a previous study using rectal cancer tissues, the high expression of PD-L1 in immune cells was associated with better prognosis, but tumor cell PD-L1 expression was not independently prognostic (29). Another study on non-small cell lung cancer demonstrated that high levels of PD-L1 expression in tumor-infiltrating macrophages correlated with “hot” tumor status (25). Although the prognostic association of PD-L1 expression in stromal immune cells can be considered as paradoxical, we would like to highlight that the function of anti-tumor immunity is regulated by a dynamic balance between stimulatory and inhibitory forces; the up-shift responses can be a trigger for the immune checkpoint molecules (24). For example, the well-known inflammatory cytokine IFN-γ is a landmark mediator of pro-inflammatory immune responses, but its function has double-edged characters in determining pro- and anti-tumor immunity (30). Taken together, the related inducers are elevated in the pro-inflammatory tumor mileu, and the underlying “hot tumor” conditions contribute to induce the PD-L1 expression in tumor-infiltrating immune cells. The expression levels of immunologic markers should be comprehensively analyzed in the tumor and stromal areas to assess the status of the tumor-immune microenvironment.

Based on the significant associations observed in the discrete analyses, we established combined sets including three immune-related variables: MDSCs, PD-1^+^/CD8^+^ TIL ratio, and PD-L1 expression in either immune cells or tumor cells. The PD-1^+^/CD8^+^ TIL ratio refers to the relativeness of PD-1-related immune-exhaustion to cytotoxic activity of effector cells. The positive correlation between MDSCs and the PD-1^+^/CD8^+^ TIL ratio, associated with worse prognosis, demonstrated the potential of PD-1^+^/CD8^+^ TIL ratio as a biomarker of the immunosuppressive tumor environment. The c-index values obtained from the two models were comparable and their prognostic strengths were acceptable for both DFS and OS. However, further external validation is required for practical application.

This study is not free of limitations. Due to the retrospective study design, inherent selection bias cannot be eliminated. Although TMAs used in this study included representative cores of tumor tissues, the use of TMAs cannot fully reflect the whole-section specimens, as intratumoral heterogeneity and dynamic characteristics of cancer immunity could not be fully presented. As the fluorescence microscope used in the study could not evaluate the expression of cytokeratin due to the limited number of markers, the specific location, e.g. intratumoral or stromal, within the tumor could not be considered in the evaluation of MDSCs. Nevertheless, to minimize the contamination of tumor cell from the surrounding normal tissue and vice versa, representative tumor tissue areas were precisely selected by two specialized pathologists. The combined effects of MDSCs and the PD-1/PD-L1 axis were analyzed by integrating long-term outcome data with IHC and IF staining results.

## Conclusion

This study demonstrated the prognostic implications of tumor-infiltrating MDSCs, PD-1^+^/CD8^+^ TIL ratio, and PD-L1 expression in stromal immune cells and tumor cells in patients with locally advanced rectal cancer. Integrating the prognostic associations of MDSCs and the PD-1/PD-L1 axis, the patients were stratified according to the combined risk levels, independent from other clinicopathological variables. The results of prognostic stratification identified a subset of patients with dismal prognosis, where the potential benefits of the combined inhibition of MDSCs and the PD-1/PD-L1 axis may be derived. Therefore, current analysis provides initial insights for ongoing or future clinical trials to target these components contributing to the immune-inhibitory tumor microenvironment. Further independent external validation is needed to introduce the generated risk assessment model to clinical practice.

## Data availability statement

The original contributions presented in the study are included in the article/**Supplementary Material**. Further inquiries can be directed to the corresponding author.

## Ethics statement

The studies involving human participants were reviewed and approved by Seoul National University Hospital. Written informed consent for participation was not required for this study in accordance with the national legislation and the institutional requirements.

## Author contributions

Conceptualization: YL and EC. Methodology: YL, JK, and MC. Formal analysis: YL, JK, MC, and SK. Investigation: YL, JK, MC, SK, and EC. Resources: YL and EC. Data curation: YL, JK, and SK. Writing: YL, JK, MC, and EC. Supervision: EC. Funding acquisition: YL and EC. All authors contributed to the article and approved the submitted version.

## Funding

This study was supported by the National Research Foundation of Korea (NRF) grant funded by the Korean government (Ministry of Science and ICT) (2021R1C1C1005297 for YL and 2020R1F1A1073616 for EC).

## Conflict of interest

The authors declare that the research was conducted in the absence of any commercial or financial relationships that could be construed as a potential conflict of interest.

## Publisher’s note

All claims expressed in this article are solely those of the authors and do not necessarily represent those of their affiliated organizations, or those of the publisher, the editors and the reviewers. Any product that may be evaluated in this article, or claim that may be made by its manufacturer, is not guaranteed or endorsed by the publisher.

## Supplementary material

Supplementary Figure 1 Overall survival curves according to high and low expression levels of **(A)** MDSCs, **(B)** PD-1^+^/CD8^+^ TIL ratio, **(C)** PD-L1 immune cell score, and **(D)** PD-L1 H-score. MDSC, myeloid-derived suppressor cell; PD-1, programmed cell death-1; TIL, tumor-infiltrating lymphocyte; PD-L1, programmed death-ligand 1.
