# Supplementary material for: Prognostic stratification based on the levels of tumor-infiltrating myeloid-derived suppressor cells and PD-1/PD-L1 axis in locally advanced rectal cancer
Source: Front Oncol. 2022 Oct 25;12:1018700. doi: 10.3389/fonc.2022.1018700 (PMC9641101; doi:10.3389/fonc.2022.1018700)
Supplement: Supplementary file 2 [file DataSheet_2.docx]

**Supplementary Table 1.** Patient, tumor, and treatment-related characteristics of the patients with rectal cancer undergoing neoadjuvant chemoradiotherapy followed by surgery

| Characteristics | Patients (%) |
| --- | --- |
| Age (years) |  |
| Median (range) | 62 (29−86) |
| Sex |  |
| Male | 116 (70) |
| Female | 49 (30) |
| Distance from anal verge (cm) |  |
| Median (range) | 5 (0−12) |
| Tumor grade at biopsy |  |
| I | 20 (12) |
| II | 143 (86) |
| III | 1 (1) |
| Unknown | 1 (1) |
| cT stage |  |
| T2 | 14 (9) |
| T3 | 139 (84) |
| T4 | 12 (7) |
| cN stage |  |
| N0 | 26 (16) |
| N1 | 121 (73) |
| N2 | 18 (11) |
| ypT stage |  |
| T0 | 31 (19) |
| T1 | 13 (8) |
| T2 | 47 (28) |
| T3 | 74 (45) |
| ypN stage |  |
| N0 | 127 (77) |
| N1 | 29 (18) |
| N2 | 8 (5) |
| Not reported | 1 (0) |
| Dworak TRG |  |
| 3–4 | 40 (24) |
| 2 | 59 (36) |
| 0–1 | 62 (38) |
| Unknown | 4 (2) |
| Lymphatic invasion |  |
| Negative | 119 (72) |
| Positive | 20 (12) |
| Unknown | 26 (16) |
| Vascular invasion |  |
| Negative | 133 (80) |
| Positive | 6 (4) |
| Unknown | 26 (16) |
| Perineural invasion |  |
| Negative | 124 (75) |
| Positive | 15 (9) |
| Unknown | 26 (16) |
| Circumferential resection margin |  |
| > 1 mm | 123 (74) |
| ≤ 1 mm | 8 (5) |
| Unknown | 34 (21) |
| Pre-RT CEA (ng/mL) |  |
| Median (range) | 2.3 (0.0−300.0) |
| Post-RT CEA (ng/mL) |  |
| Median (range) | 1.6 (0.0−42.9) |
| Postoperative CEA (ng/mL) |  |
| Median (range) | 1.1 (0.0−7.2) |
| Microsatellite instability |  |
| MSS | 104 (63) |
| MSI-L or MSI-H | 10 (6) |
| Unknown | 51 (31) |
| RT-Op interval (days) |  |
| Median (range) | 53 (38–111) |
| Types of surgery |  |
| Sphincter-preserving | 156 (94) |
| Abdominoperineal resection | 9 (6) |
| Adjuvant chemotherapy |  |
| Yes | 149 (90) |
| No | 16 (10) |

TRG, tumor regression grade; CEA, carcinoembryonic antigen; MSS, microsatellite stable; MSI-L, microsatellite instability-low; MSI-H, microsatellite instability-high; RT, radiotherapy; Op, operation.

**Supplementary Table 2.** Levels of MDSCs, PD-1^+^/CD8^+^ TIL ratio, PD-L1 immune cell score, and PD-L1 H-score

| Immune-related markers^*^ | Patients (%) |
| --- | --- |
| MDSCs |  |
| Low | 70 (42) |
| High | 89 (54) |
| Unknown | 6 (4) |
| PD-1^+^/CD8^+^ TIL ratio |  |
| Low | 127 (77) |
| High | 18 (11) |
| Unknown | 20 (12) |
| PD-L1 immune cell score (stromal) |  |
| Low | 25 (15) |
| High | 105 (64) |
| Unknown | 35 (21) |
| PD-L1 H-score (tumor) |  |
| Low | 117 (71) |
| High | 14 (8) |
| Unknown | 34 (21) |

MDSC, myeloid-derived suppressor cell; PD-1, programmed cell death-1; TIL, tumor-infiltrating lymphocyte; PD-L1, programmed death-ligand 1.

^*^The cutoff values: < 3 vs. ≥ 3, ≤ 0.1725 vs. > 0.1725, ≤ 20 vs. > 20, and ≤ 200 vs. > 200 for the low vs. high levels of MDSCs, PD-1^+^/CD8^+^ TIL ratio, stromal PD-L1 immune cell score, and PD-L1 H-score, respectively.

**Supplementary Table 3.** Relationships between immune cell-related expression levels and clinicopathological factors

| Variables | | MDSCs | | | PD-1^+^/CD8^+^ TIL ratio | | | PD-L1 immune cell score | | | PD-L1 H-score | | |
| --- | --- | --- | --- | --- | --- | --- | --- | --- | --- | --- | --- | --- | --- |
|  |  | Low  (n = 70) | High  (n = 89) | *P* | Low  (n = 127) | High  (n = 18) | *P* | Low  (n = 25) | High  (n = 105) | *P* | Low  (n = 117) | High  (n = 14) | *P* |
| Age  (years) | ≤ 62 | 41 (59) | 38 (43) | 0.047 | 67 (53) | 6 (33) | 0.123 | 12 (48) | 55 (52) | 0.694 | 60 (51) | 5 (36) | 0.271 |
|  | > 62 | 29 (41) | 51 (57) |  | 60 (47) | 12 (67) |  | 13 (52) | 50 (48) |  | 57 (49) | 9 (64) |  |
| Sex | Female | 19 (27) | 29 (33) | 0.458 | 36 (28) | 8 (44) | 0.164 | 6 (24) | 34 (32) | 0.415 | 36 (31) | 4 (29) | 1.000 |
|  | Male | 51 (73) | 60 (67) |  | 91 (72) | 10 (56) |  | 19 (76) | 71 (68) |  | 81 (69) | 10 (71) |  |
| Distance from  anal verge (cm) | > 5 | 30 (43) | 37 (42) | 0.871 | 53 (42) | 8 (44) | 0.827 | 10 (40) | 45 (43) | 0.795 | 48 (41) | 7 (50) | 0.520 |
|  | ≤ 5 | 40 (57) | 52 (58) |  | 74 (58) | 10 (56) |  | 15 (60) | 60 57) |  | 69 (59) | 7 (50) |  |
| Tumor grade  (biopsy) | I | 7 (10) | 13 (15) | 0.370 | 17 (13) | 2 (11) | 1.000 | 4 (16) | 14 (13) | 0.750 | 16 (14) | 2 (14) | 1.000 |
|  | II–III | 63 (90) | 75 (85) |  | 110 (87) | 16 (89) |  | 21 (84) | 91 (87) |  | 101 (86) | 12 (86) |  |
| cT stage | T2 | 5 (7) | 9 (10) | 0.512 | 11 (9) | 2 (11) | 0.665 | 1 (4) | 6 (6) | 1.000 | 6 (5) | 1 (7) | 0.556 |
|  | ≥ T3 | 65 (93) | 80 (90) |  | 116 (91) | 16 (89) |  | 24 (96) | 99 (94) |  | 111 (95) | 13 (93) |  |
| cN stage | N0 | 14 (20) | 11 (12) | 0.189 | 16 (13) | 1 (6) | 0.696 | 3 (12) | 14 (13) | 1.000 | 18 (15) | 2 (14) | 1.000 |
|  | N+ | 56 (80) | 78 (88) |  | 111 (87) | 17 (94) |  | 22 (88) | 91 (87) |  | 99 (85) | 12 (86) |  |
| ypT stage | T0–2 | 36 (51) | 51 (57) | 0.460 | 70 (55) | 12 (67) | 0.355 | 8 (32) | 53 (51) | 0.096 | 59 (50) | 2 (14) | 0.011 |
|  | ≥ T3 | 34 (49) | 38 (43) |  | 57 (45) | 6 (33) |  | 17 (68) | 52 (49) |  | 58 (50) | 12 (86) |  |
| ypN stage | N0 | 56 (80) | 67 (76) | 0.561 | 97 (77) | 15 (83) | 0.764 | 15 (63) | 79 (75) | 0.205 | 90 (77) | 5 (38) | 0.003 |
|  | N1–2 | 14 (20) | 21 (24) |  | 29 (23) | 3 (17) |  | 9 (37) | 26 (25) |  | 27 (23) | 8 (62) |  |
| Downstaging  of T | Yes | 35 (51) | 53 (62) | 0.173 | 69 (56) | 12 (75) | 0.182 | 8 (32) | 53 (53) | 0.060 | 58 (52) | 3 (21) | 0.046 |
|  | No | 34 (49) | 33 (38) |  | 55 (44) | 4 (25) |  | 17 (68) | 47 (47) |  | 54 (48) | 11 (79) |  |
| Downstaging  of N | Yes | 48 (69) | 59 (67) | 0.839 | 89 (71) | 14 (78) | 0.781 | 14 (58) | 71 (68) | 0.387 | 79 (68) | 4 (31) | 0.014 |
|  | No | 22 (31) | 29 (33) |  | 37 (29) | 4 (22) |  | 10 (42) | 34 (32) |  | 38 (32) | 9 (69) |  |
| pCR | Yes | 14 (20) | 12 (14) | 0.294 | 22 (18) | 4 (25) | 0.498 | 1 (4) | 0 (0) | 0.200 | 2 (2) | 0 (0) | 1.000 |
|  | No | 55 (80) | 74 (86) |  | 102 (82) | 12 (75) |  | 24 (96) | 100 (100) |  | 110 (98) | 14 (100) |  |
| Dworak TRG | 3–4 | 22 (32) | 16 (18) | 0.027 | 34 (27) | 5 (28) | 0.474 | 3 (13) | 8 (8) | 0.558 | 11 (10) | 0 (0) | 0.053 |
|  | 2 | 28 (41) | 29 (34) |  | 44 (36) | 4 (22) |  | 8 (33) | 45 (44) |  | 51 (45) | 3 (21) |  |
|  | 0–1 | 19 (27) | 41 (48) |  | 46 (37) | 9 (50) |  | 13 (54) | 49 (48) |  | 51 (45) | 11 (79) |  |
| Lymphatic invasion | No | 48 (86) | 66 (86) | 1.000 | 89 (85) | 13 (93) | 0.689 | 18 (75) | 91 (87) | 0.154 | 101 (88) | 8 (57) | 0.003 |
|  | Yes | 8 (14) | 11 (14) |  | 16 (15) | 1 (7) |  | 6 (25) | 14 (13) |  | 14 (12) | 6 (43) |  |
| Vascular invasion | No | 54 (96) | 73 (95) | 1.000 | 100 (95) | 13 (93) | 0.536 | 22 (92) | 101 (96) | 0.310 | 110 (96) | 13 (93) | 0.505 |
|  | Yes | 2 (4) | 4 (5) |  | 5 (5) | 1 (7) |  | 2 (8) | 4 (4) |  | 5 (4) | 1 (7) |  |
| Perineural invasion | No | 48 (86) | 71 (92) | 0.228 | 92 (88) | 14 (100) | 0.360 | 20 (83) | 94 (89) | 0.478 | 104 (90) | 10 (71) | 0.059 |
|  | Yes | 8 (14) | 6 (8) |  | 13 (12) | 0 (0) |  | 4 (17) | 11 (11) |  | 11 (10) | 4 (29) |  |
| CRM (mm) | > 1 | 49 (93) | 68 (94) | 0.721 | 96 (96) | 11 (85) | 0.141 | 22 (96) | 92 (93) | 1.000 | 101 (94) | 12 (92) | 1.000 |
|  | ≤ 1 | 4 (7) | 4 (6) |  | 4 (4) | 2 (15) |  | 1 (4) | 7 (7) |  | 7 (6) | 1 (8) |  |

MDSC, myeloid-derived suppressor cell; PD-1, programmed cell death-1; TIL, tumor-infiltrating lymphocyte; PD-L1, programmed death-ligand 1; pCR, pathologic complete response; TRG, tumor regression grade; CRM, circumferential resection margin; CRT, chemoradiotherapy; CEA, carcinoembryonic antigen; RT, radiotherapy; Op, operation.

^*^The cutoff values: < 3 vs. ≥ 3, ≤ 0.1725 vs. > 0.1725, ≤ 20 vs. > 20, and ≤ 200 vs. > 200 for the low vs. high levels of MDSCs, PD-1^+^/CD8^+^ TIL ratio, stromal PD-L1 immune cell score, and PD-L1 H-score, respectively.

**Supplementary Table 4.** Univariate analysis of prognostic factors

| Variables | Disease-free survival | | Overall survival | |
| --- | --- | --- | --- | --- |
|  | 7-year rates (%) | *P* | 7-year rates (%) | *P* |
| Age (years) |  |  |  |  |
| ≤ 62 | 71 | 0.369 | 82 | 0.032 |
| > 62 | 70 |  | 75 |  |
| Sex |  |  |  |  |
| Female | 77 | 0.215 | 85 | 0.137 |
| Male | 68 |  | 75 |  |
| Distance from AV |  |  |  |  |
| > 5 cm | 75 | 0.285 | 83 | 0.155 |
| ≤ 5 cm | 68 |  | 75 |  |
| Tumor grade at biopsy |  |  |  |  |
| I | 57 | 0.515 | 76 | 0.609 |
| II–III | 72 |  | 78 |  |
| cT stage |  |  |  |  |
| T2 | 79 | 0.562 | 93 | 0.212 |
| ≥ T3 | 70 |  | 77 |  |
| cN stage |  |  |  |  |
| N0 | 77 | 0.229 | 84 | 0.189 |
| N+ | 70 |  | 77 |  |
| ypT stage |  |  |  |  |
| T0–2 | 80 | 0.002 | 86 | 0.009 |
| ≥ T3 | 59 |  | 68 |  |
| ypN stage |  |  |  |  |
| N0 | 77 | 0.003 | 84 | 0.002 |
| N1–2 | 51 |  | 59 |  |
| pCR |  |  |  |  |
| Yes | 82 | 0.115 | 82 | 0.487 |
| No | 67 |  | 77 |  |
| Dworak TRG |  |  |  |  |
| 3–4 | 87 | < 0.001 | 87 | 0.012 |
| 2 | 78 |  | 83 |  |
| 0–1 | 53 |  | 67 |  |
| Lymphatic invasion |  |  |  |  |
| No | 72 | 0.001 | 80 | 0.017 |
| Yes | 45 |  | 60 |  |
| Vascular invasion |  |  |  |  |
| No | 69 | 0.146 | 78 | 0.453 |
| Yes | 50 |  | 67 |  |
| Perineural invasion |  |  |  |  |
| No | 72 | 0.007 | 82 | < 0.001 |
| Yes | 40 |  | 37 |  |
| CRM status |  |  |  |  |
| > 1 mm | 71 | 0.273 | 78 | 0.345 |
| ≤ 1 mm | 50 |  | 63 |  |
| Pre-RT CEA |  |  |  |  |
| Low | 84 | 0.010 | 92 | 0.003 |
| High | 64 |  | 72 |  |
| Post-RT CEA |  |  |  |  |
| Low | 78 | 0.007 | 85 | 0.005 |
| High | 58 |  | 66 |  |
| Post-Op CEA |  |  |  |  |
| Low | 75 | 0.009 | 81 | 0.070 |
| High | 60 |  | 71 |  |
| Types of surgery |  |  |  |  |
| Sphincter-preserving | 72 | 0.048 | 80 | 0.055 |
| APR | 44 |  | 56 |  |
| Adjuvant chemotherapy |  |  |  |  |
| Yes | 71 | 0.660 | 78 | 0.513 |
| No | 69 |  | 81 |  |
| MDSCs |  |  |  |  |
| Low | 84 | < 0.001 | 88 | 0.001 |
| High | 59 |  | 70 |  |
| PD-1^+^/CD8^+^ TIL ratio |  |  |  |  |
| Low | 71 | 0.042 | 79 | 0.046 |
| High | 56 |  | 67 |  |
| PD-L1 immune cell score |  |  |  |  |
| Low | 52 | 0.047 | 64 | 0.117 |
| High | 70 |  | 80 |  |
| PD-L1 H-score |  |  |  |  |
| Low | 72 | < 0.001 | 79 | 0.001 |
| High | 21 |  | 50 |  |
| [Set I] MDSC^High^, PD-1^+^/CD8^+High^,  and PD-L1 immune cell score^Low^ |  |  |  |  |
| None | 86 | < 0.001 | 91 | < 0.001 |
| 1 risk factor | 64 |  | 78 |  |
| 2‒3 risk factors | 35 |  | 48 |  |
| [Set II] MDSC^High^, PD-1^+^/CD8^+High^,  and PD-L1 H-score^High^ |  |  |  |  |
| None | 84 | < 0.001 | 88 | 0.001 |
| 1 risk factor | 62 |  | 74 |  |
| 2‒3 risk factors | 30 |  | 55 |  |

AV, anal verge; CR, complete response; TRG, tumor regression grade; CRM, circumferential resection margin; RT, radiotherapy; CEA, carcinoembryonic antigen; Op, operation; APR, abdominoperineal resection; MDSC, myeloid-derived suppressor cell; PD-1, programmed cell death-1; TIL, tumor-infiltrating lymphocyte; PD-L1, programmed death-ligand 1.
