# Supplementary figures and images for: Prognostic stratification based on the levels of tumor-infiltrating myeloid-derived suppressor cells and PD-1/PD-L1 axis in locally advanced rectal cancer
Source: Front Oncol. 2022 Oct 25;12:1018700. doi: 10.3389/fonc.2022.1018700 (PMC9641101; doi:10.3389/fonc.2022.1018700)

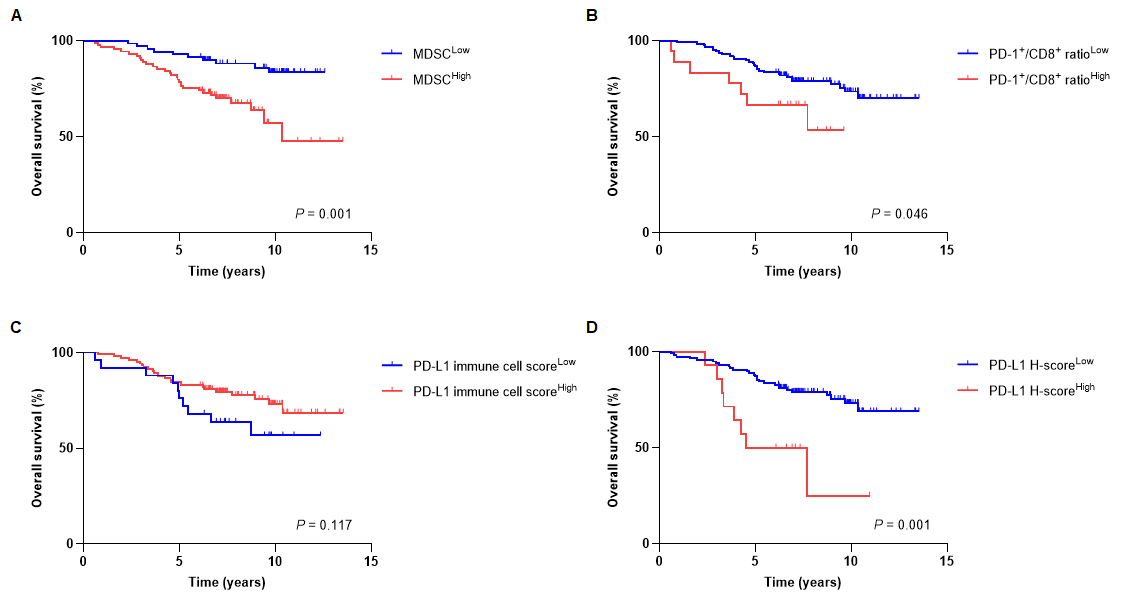

Supplement: Supplementary Figure 1 — Overall survival curves according to high and low expression levels of (A) MDSCs, (B) PD-1+/CD8+ TIL ratio, (C) PD-L1 immune cell score, and (D) PD-L1 H-score. MDSC, myeloid-derived suppressor cell; PD-1, programmed cell death-1; TIL, tumor-infiltrating lymphocyte; PD-L1, programmed death-ligand 1. [file Image_1.tif]
